# Advocacy and policy leadership training for primary care: a global environmental scan of competency frameworks, interventions, and strategic priorities

**DOI:** 10.3389/fmed.2026.1894165

**Published:** 2026-09-30

**Authors:** Anis Kazi, Ellena Andoniou, Jamie Rodas, Praseedha Janakiram, Katherine Dominique Rouleau

**Affiliations:** 1 Department of Family and Community Medicine, Temerty Faculty of Medicine, University of Toronto, Toronto, ON, Canada; 2 The Institute of Education Research, University Health Network, Toronto, ON, Canada

**Keywords:** family medicine, primary health care, health advocacy, health policy, leadership, competency-based education, graduate medical education, low- and middle-income countries

## Abstract

**Background:**

Primary health care (PHC) strengthening globally is catalysed by family physicians and primary care professionals equipped with integrated advocacy, policy leadership, and governance competencies. Yet training in these domains remains fragmented and inconsistent across both high-income countries (HICs) and low- and middle-income countries (LMICs), limiting primary care providers’ capacity to influence health systems, advance equity, and engage in the policy processes essential to PHC system strengthening and universal health coverage (UHC). International academic partnerships aimed at PHC transformation require a shared, rigorous evidence base to inform collaborative curriculum co-design across contexts.

**Methods:**

An environmental scan of global evidence was conducted in December 2025 to map advocacy and policy leadership frameworks, competencies, and educational interventions for primary care professionals. A scoping search of PubMed, Scopus, ERIC, and Google Scholar, supplemented by grey literature, identified 176 potentially relevant papers (1976–2025). After title, abstract, and full-text screening, 20 core sources published between 2004 and 2024 were selected for detailed structured extraction using a standardized 32-field template across three analytical lenses: conceptual frameworks, competency definitions, and educational interventions. Thematic synthesis was organized around seven major themes aligned with PHC system-strengthening priorities.

**Results:**

Four foundational competency frameworks were identified: the CanMEDS Health Advocate role; the South African District Health System Family Physician Leadership and Governance Framework; the Turkish Family Medicine Health Advocacy Competency Set (derived through Delphi consensus); and Kuehne et al.'s PHC Provider Advocacy Framework. All four converge on six core competency domains: health policy literacy, communication, community engagement, equity focus, leadership and governance, and interprofessional collaboration. Educational interventions ranged from brief didactic sessions (approximately 10% of programs) to longitudinal, experiential curricula (approximately 40%). Evidence for effectiveness was strongest at the learner-level improved knowledge and attitudes documented across 75% of postgraduate programs, but evidence for sustained behavioural change and organizational or policy-level system impacts was sparse, particularly in Low- and Middle-Income Countries (LMIC) settings. The evidence base was predominantly High-Income country (HIC)-focused (55%), with only three sources explicitly developing LMIC-adapted frameworks. Major gaps include the near-absence of evaluated LMIC-specific postgraduate curricula, virtually no studies linking training to health system policy changes, and limited attention to advocacy ethics and safety in politically constrained environments.

**Conclusion:**

The global evidence base supports competency-based, longitudinal, and experiential approaches to advocacy and policy leadership training for primary care but is critically underdeveloped for LMIC and district health system contexts. Eight strategic priorities are proposed and centered on: designing context-adapted curricula within international collaborative frameworks; establishing academic networks for mentorship and resource sharing; conducting rigorous longitudinal outcome studies; and integrating advocacy and policy literacy into accreditation standards globally. These priorities are framed as the evidence-informed foundation for reciprocal North–South and South–South academic partnerships to advance PHC transformation, health equity, and UHC.

## 1 Introduction

Primary health care (PHC) has been internationally recognized since the 1978 Alma-Ata Declaration as the foundation of equitable, effective, and sustainable health systems (1). This commitment was reaffirmed through the 2018 Astana Declaration and ongoing World Health Organization (WHO) strategies explicitly linking PHC to Universal Health Coverage (UHC), health equity, and population health improvement through principles of accessibility, comprehensiveness, person-centred care, and multi-sectoral engagement (2, 3). Across both HICs and LMICs, PHC is increasingly positioned as central to health system resilience, continuity of care, and responsiveness to population needs, a reality underscored by the COVID-19 pandemic which highlighted the critical importance of resilient PHC infrastructure and workforce development (4–6). Yet despite this policy consensus, PHC-oriented health systems globally remain fragile, underinvested, and unequally distributed, with persistent implementation gaps across all income settings (3, 7).

Family medicine has emerged as a cornerstone of PHC-oriented system strengthening. Family physicians bring clinical continuity, person-centred care, and community orientation to primary care service delivery, and are uniquely positioned at the interface of individuals, communities, and health systems (5, 7). Beyond clinical work, family physicians are increasingly expected to take on leadership roles in district health system governance, workforce capacity building, and health advocacy at local, national, and policy levels (5, 8). In many HICs, primary care faces challenges including fragmentation, workforce shortages, uneven access, and limited integration with public health and social services (9). In LMICs, these challenges are compounded by constrained fiscal space, donor-driven priorities, evolving governance structures, and limited representation of primary care professionals in health policy decision-making (10, 11). Effective PHC strengthening therefore depends not only on ensuring the training and education of a sufficient primary care workforce, but on building physicians’ capacity to engage meaningfully with health systems, advocate for equitable policies, and exercise leadership within complex governance environments (5, 12). Indeed, while the widely called-for transition to multi-disciplinary primary care teams ultimately requires leadership from a range of primary care workers, the leadership role of the primary care physicians has been repeatedly underscored. However, the formal development of advocacy and policy leadership competencies within family medicine training remains inconsistent globally (13). While some postgraduate programs in both HICs and LMICs have introduced explicit advocacy curricula, this integration remains shallow and uneven with health system priorities and vary widely (14–16). In most postgraduate family medicine training programs, where such training exist at all, have not systematically integrated advocacy, policy literacy, and leadership competencies, and where such training exists, it is often brief, didactic, and disconnected from actual governance structures or community engagement opportunities (17, 18). As a result, many family physicians enter practice without structured preparation for policy engagement or systems-level advocacy, despite growing expectations that they assume such roles (19). In a national survey of U.S. family medicine residency programs, only approximately one-third reported a mandatory advocacy curriculum, with curricular flexibility and faculty capacity cited as the primary barriers (20). This gap is particularly acute in LMIC contexts, where district health systems rely heavily on family physicians as leaders, advocates, and capacity builders, yet where formal training in these domains remains largely *ad hoc* or absent (8, 18).

The challenge is further compounded by conceptual ambiguity. In the medical education literature, advocacy, leadership, and policy literacy are related but distinct constructs. Health advocacy refers to action-oriented engagement aimed at promoting changes that improve health outcomes (17); leadership and governance refer to guiding and managing organizations and health systems from within (8); and policy literacy refers to understanding how health policies are formulated, financed, implemented, and evaluated, together with the capacity to translate clinical or population health evidence into policy-relevant arguments (12, 21). Recent work from PHC-oriented and LMIC contexts has begun to integrate these constructs: Kuehne et al. (12), explicitly frame advocacy as a collective responsibility for all primary care providers physicians, nurses, midwives, and community health workers to strengthen health systems and advance UHC, distinguishing between micro-level (individual patient or community), meso-level (organizational and district), and macro-level (national and global policy) advocacy. The South African district health system family physician leadership framework similarly embeds advocacy within broader leadership and governance roles at the district level, emphasizing that improving care delivery and advancing equity depend not only on advocacy for change, but on the capacity to exercise informed leadership within health systems (8, 18).

The importance of building this integrated capacity is especially acute for international academic partnerships aimed at PHC-oriented transformation, including primary care strengthening. North–South and South–South academic partnerships have emerged as critical mechanisms for sharing expertise, adapting educational models to local contexts, and building institutional capacity (22, 23). However, the effectiveness of such partnerships depends on shared conceptual frameworks, transferable educational approaches, and evidence-based guidance on what works, for whom, and under what conditions. An upcoming multi-regional, collaborative initiative bringing together academic institutions from diverse HIC and LMIC contexts to co-design advocacy and policy leadership curricula exemplifies this approach and is explicitly grounded in the evidence synthesized through the present review. This collaborative model reflects the principles of SDG 17 (Partnerships for the Goals) and positions the present environmental scan as a foundational evidence resource for equitable, reciprocal academic partnership in global PHC capacity building.

Existing systematic reviews of advocacy training in medical education consistently find positive learner-level outcomes but also highlight critical limitations: nearly all evaluated programs focus on postgraduate training in HICs, predominantly in paediatrics and internal medicine; very few document sustained behavioural change beyond training completion; and there is virtually no evidence linking advocacy training to organizational or health system outcomes (14–16). This is unsurprising: advocacy functions as one contributing pathway among many that shape meso- and macro-level policy change and micro-level behavioural change, and is rarely, if ever, solely responsible for such outcomes. Isolating its independent effect on complex, multi-causal health system or policy outcomes is therefore inherently methodologically difficult, and this scan does not assume advocacy training alone is sufficient to produce health system change. Moreover, existing reviews do not comprehensively address PHC oriented health system-specific frameworks, do not explicitly synthesize evidence on the integration of advocacy, leadership, and policy literacy, and provide limited LMIC-inclusive co-creation opportunities with even less consideration for applications of these tools and strategies within LMIC or resource-constrained settings (21). Significant gaps therefore remain in understanding how to design, implement, and evaluate advocacy and policy leadership training aligned with PHC system-strengthening priorities, contextually adapted for diverse settings, and demonstrably contributing to PHC system-strengthening, health system performance, and equity outcomes.

This environmental scan addresses these gaps by synthesizing global evidence on advocacy and health policy leadership frameworks, competencies, and educational interventions for family physicians and other primary care professionals. Specifically, the review aims to: (1) identify and synthesize conceptual and competency frameworks for advocacy and policy leadership in primary care and PHC; (2) map and categorize educational and training interventions; (3) synthesize evidence on effectiveness and outcomes across multiple levels; (4) assess the integration of equity, community engagement, and social accountability; (5) identify barriers and enablers to implementation, particularly in LMIC contexts; and (6) propose strategic priorities for advancing the field, with particular emphasis on LMIC-oriented work and academic partnership opportunities.

## 2 Methods

### 2.1 Review design

This environmental scan was conducted in December 2025 as a rapid, integrated evidence synthesis designed to map the fragmented global landscape of advocacy and health policy leadership training for primary care clinicians. Given the scarcity of indexed literature on this topic particularly for LMIC contexts a standard systematic review methodology was adapted to include a broader range of evidence types while remaining feasible within the available timeframe. The approach comprised three linked steps: (1) scoping search and screening to identify potentially relevant conceptual frameworks, reviews, and curricula from academic and grey literature; (2) selection of a core source set judged most relevant to the review question; and (3) structured data extraction and thematic synthesis organized around predefined analytic domains aligned with PHC system-strengthening priorities.

### 2.2 Data sources and search strategy

Sources were identified through comprehensive searching of PubMed, Scopus, ERIC, and Google Scholar, with no geographic restriction applied, though the search was limited to English-language sources. Search terms combined controlled vocabulary (MeSH terms where applicable) and keywords related to: (1) population (family physicians, primary care, general practice, family medicine, PHC); (2) intervention (advocacy, health advocacy, policy, policy literacy, leadership, governance, social accountability); and (3) context (education, training, curriculum, competency, framework, residency, postgraduate). Grey literature was searched across WHO, WONCA, professional college and primary care organization websites. The scoping search, conducted between December 1 and December 15, 2025, identified 176 potentially relevant papers published between 1976 and 2025.

### 2.3 Inclusion and exclusion criteria

Sources were included if they: (1) focused on primary care or family medicine professionals, including physicians, nurses, midwives, or interprofessional teams; (2) addressed health advocacy, policy leadership, governance, or social accountability as defined competencies, roles, or training outcomes; (3) were published between 2004 and 2024; and (4) included actionable frameworks, competency definitions, or educational intervention descriptions suitable for informing curriculum design or training policy. Sources were excluded if they: (1) focused exclusively on specialties without clear transferability to PHC; (2) described advocacy solely as individual patient navigation without addressing broader health system or policy engagement; (3) provided only opinion or commentary without empirical data or framework descriptions; or (4) were published before 2004, predating the formalization of advocacy as a competency domain in major medical education frameworks.

### 2.4 Selection and extraction

From approximately 50 sources retained after full-text screening, 20 core sources were selected for detailed extraction, prioritizing sources that described primary care- or family medicine-specific competency frameworks or educational interventions, reported on diverse geographic contexts, addressed multiple advocacy levels (micro, meso, macro), and included outcome data or evaluation findings. The final core set comprised 13 peer-reviewed articles (65%), 4 framework descriptions from professional organizations or consensus processes (20%), 2 grey literature reports or program evaluations (10%), and 1 educational program description (5%). A manual data extraction protocol using a standardized 32-field template was applied across three analytical lenses (Outcomes, Concepts, Pedagogy), and all data were entered into a master evidence table. Key fields achieved 100% data completeness across all 20 sources.

### 2.5 Synthesis and quality assurance

Thematic synthesis was organized around seven major themes: (1) definitions and conceptualizations of advocacy; (2) competency frameworks; (3) educational and training interventions; (4) evidence of effectiveness organized by Kirkpatrick levels; (5) equity, community engagement, and social accountability; (6) barriers and enablers; and (7) identified gaps and future priorities. Synthesis followed an iterative process of initial coding, organization into higher-level analytic categories, and cross-case comparison. Every claim is traceable to a specific source in the Master Evidence Table. All structured data have been cross-checked against original full texts to ensure accuracy and internal consistency.

## 3 Results

### 3.1 Characteristics of the evidence base

The master evidence table comprised of 20 unique sources published between 2004 and 2024, drawn from a broader scoping set of approximately 50 sources. Publication density increased markedly from 2019 onward, reflecting growing interest in competency-based approaches to advocacy training. Table 1 summarizes the distribution of sources across geographic focus, specialty, and training level.

**Table 1:** Distribution of core sources by geographic focus, specialty, and training level (*N* = 20).

Dimension	Category	*N* (%)	Notable examples/notes
Geographic Focus	HIC-focused	11 (55%)	Canada 7 (Howell 2019; McDonald 2019; Bernard 2019; Bhate 2015; Law 2016; Pottie 2007; Kates 2012); USA 3; Europe 1 (14, 21, 25–29)
	Global/Multi-country	10 (50%)	Kuehne 2022; Rouleau 2018; van Weel 2004; Ponka 2020; Amor 2022 (5, 12, 23, 30, 31)
	LMIC-specific elements	3 (15%)	South Africa (Mash 2016; von Pressentin 2024); Turkey (Demirören 2024); explicit global PHC framework (Kuehne 2022) (8, 12, 18, 24)
Specialty Focus	Family Medicine-specific	5 (25%)	Mash 2016; von Pressentin 2024; Demirören 2024; Bernard 2019; Coutinho 2020 (8, 18, 20, 24, 25)
	Mixed health professionals	5 (25%)	Interprofessional advocacy initiatives with physicians, nurses, midwives, CHWs
	Paediatrics (PHC-adjacent)	5 (25%)	Predominantly North American GME; Howell 2019; Scott 2019; Chamberlain 2013 (14, 15, 32)
	Nursing/Midwifery/APN	3 (15%)	Heinen 2019; Dawson 2015; Langins 2016 (33–35)
	Health policy/administration	2 (10%)	Schleiff 2020; White 2015 (36, 37)
Training Level	Postgraduate/GME residency	15 (75%)	Dominant context in the published evidence base
	Undergraduate/medical student	2 (10%)	Bhate 2015; Pottie 2007 (26, 28)
	CPD/continuing education	2 (10%)	Amor 2022; Lalonde 2009 (31, 38)
	Mixed/unspecified	1 (5%)	

The evidence base was predominantly HIC-focused: 11 sources (55%) addressed HIC settings (Canada: 7 sources; USA: 3 sources; Europe: 1 source). Ten sources (50%) were global or multi-country in framing. No sources focused exclusively on LMICs, though three explicitly integrated LMIC contexts: the South African district health system framework (8, 18), the Turkish family medicine Delphi study (24), and them (12), global PHC advocacy framework. By specialty, the evidence base included five family medicine-specific sources (25%), five mixed health professional sources (25%), five paediatrics sources (25%), three nursing/midwifery/advanced practice sources (15%), and two health policy or administration sources (10%). By educational level, 15 sources (75%) addressed postgraduate or Graduate Medical Education (GME) residency training, two (10%) addressed undergraduate or medical student education, two (10%) addressed Continuing Professional Development (CPD) or continuing education, and one (5%) was mixed or unspecified.

### 3.2 Definitions and conceptualizations of advocacy in primary care and PHC

#### 3.2.1 From general physician advocacy to PHC-specific framing

The definition of health advocacy in the medical education literature has evolved over two decades from a values-based aspiration to an operationalized competency domain. The foundational definition articulated by Earnest et al. (17) characterizes physician advocacy as “action by a physician to promote social, economic, educational, and political changes to improve the health of individuals, communities, and populations,” explicitly encompassing multiple levels and addressing social determinants of health, equity, and justice (17). The CanMEDS framework institutionalized advocacy as a core physician role, defining the Health Advocate as one who “responds to individuals” health needs and advocates with them for improved health and health care” (19, 39). While this elevated advocacy's status in medical professionalism, the framing initially remained generic and still predominantly “individual” focused without explicitly addressing health systems, policy processes, or PHC-specific dimensions, a limitation acknowledged within the framework itself (19).

More recent work from PHC-oriented and LMIC contexts has reframed advocacy as a responsibility of PHC-committed providers specifically designed to strengthen health systems and advance UHC. Drawing on the conceptual architecture of the Primary Health Care Performance Initiative (PHCPI) (40), Kuehne et al. (12), distinguish between: micro-level advocacy (addressing individual patient or community health needs, such as navigating health services or accessing medicines); meso-level advocacy (influencing organizational and district health system decisions, including clinic governance, health facility planning, and human resource allocation); and macro-level advocacy (influencing national health policy and global agendas through health financing reform, legislative advocacy, and health system equity policies). These PHC-specific frameworks recognize that primary care professionals, working at the interface of clinics and communities, are uniquely positioned to engage across all three levels, and that building this multilevel advocacy capacity is essential to PHC system strengthening.

#### 3.2.2 Advocacy, leadership, and policy literacy as interdependent constructs

The reviewed literature clarifies that advocacy, leadership, and policy literacy while analytically distinct, function as interdependent competencies in PHC-oriented practice. HIC curricula, predominantly from paediatrics and internal medicine, tend to conceptualize advocacy as communication and activism (legislative visits, media engagement, community organizing, patient advocacy), often treating it as a discrete skill set separate from formal leadership or in-depth policy analysis (14, 15, 17). In contrast, literature from PHC-oriented and LMIC contexts particularly the South African family physician leadership framework and the Kuehne PHC advocacy framework positions advocacy, leadership, and policy literacy as mutually reinforcing competencies embedded within governance roles at the district or system level (8, 12, 18). As synthesized in Supplementary Appendix B, this integrated framing reflects the realities of PHC system strengthening, where improving care delivery and advancing equity depend not only on advocacy for change but on the capacity to understand policy environments and exercise leadership within health systems. Evidence from the scoping review suggests that advocacy training most likely to produce sustained behavioural change is that which explicitly integrates all three domains in a coordinated and contextually anchored manner (18, 21).

### 3.3 Advocacy and policy leadership competency frameworks

#### 3.3.1 Overview and comparative analysis

Synthesis across the evidence base identified four foundational competency frameworks that most directly inform advocacy and policy leadership training for family physicians and primary care professionals. These are described below and compared in Table 2 (summary) and Supplementary Appendix A (full comparison) and Supplementary Appendix B (detailed descriptions and implementation examples).

**Table 2:** Comparative summary of core competency domains across major frameworks (see Supplementary Appendix B for full comparison).

Competency domain	CanMEDS (19)	South Africa (8)	Turkey (24)	Kuehne PHC (12)
Health policy literacy	Partial—implicit	Explicit—district systems, financing	Explicit—system context	Explicit—policy cycles, financing, governance
Communication & media	Implicit	Explicit	Explicit	Explicit
Community engagement & organizing	Partial	Explicit—COPC role (core FP role)	Explicit	Explicit
Equity focus & social determinants	Partial	Explicit—values-based leadership	Explicit	Explicit—equity and social accountability
Leadership & governance	Partial—across multiple roles	Explicit—core organizing principle	Moderate	Strong—collective, system-level action
Interprofessional collaboration	Partial	Explicit	Partial	Explicit—PHC teams and CHWs
Advocacy ethics & safety	Absent	Partial	Partial	Partial
LMIC/district health system context	Absent	Explicit—South Africa	Explicit—Turkey	Explicit—global LMIC
Degree of operationalization	Low	High	Moderate–High	Moderate

The CanMEDS Health Advocate Role (19, 39) is the most widely adopted generic cross-specialty framework, defining four high-level advocacy roles: responding to individuals’ health needs; identifying and responding to broader determinants of health; working with communities to improve health; and contributing to health systems and policy improvement. While foundational, CanMEDS provides high-level normative statements rather than granular curriculum design or assessment guidance, and the Health Advocate role is consistently identified as among the least understood and most inconsistently taught across medical education (19). As documented in Supplementary Appendix B, its degree of operationalization is low.

The South African District Health System Family Physician Leadership and Governance Framework (8, 18) is the most elaborated family medicine-specific competency framework grounded in a health system context. Developed through a national consensus process, it defines six core roles for family physicians (clinician, consultant, capacity-builder, trainer, community-oriented primary care champion, and clinical governance leader) and employs an “I–We–It” model: I (self-awareness, reflection, personal values); We (communication, teamwork, advocacy, coordination); and It (understanding health system structure, policy environments, financing, and governance). Advocacy is explicitly embedded within the “We” domain as a core relational competency, and leadership is conceptualized as influence, system navigation, and capacity to work across organizational and community boundaries not administrative management. The framework has been operationalized through a University of Cape Town program redesign incorporating group coaching, reflective practice, and transformative learning, embedding registrars in district health teams and governance structures (18). As described in Supplementary Appendix B, this represents the most mature LMIC implementation model currently in the literature.

The Turkish Family Medicine Health Advocacy Competency Set (24) was derived through a modified Delphi process with academic and practicing family physicians to identify competencies, teaching methods, assessment approaches, and integration strategies for health advocacy in family medicine residency programs. As detailed in Supplementary Appendix B, its core competency domains span patient-centred and community-focused care, health promotion and protection, communication and ethical practice, and integration with other professional roles. Unlike high-level generic frameworks, this Delphi-derived set offers an operationalized, context-specific articulation of advocacy competencies that bridges conceptual endorsement with actionable teaching approaches and assessment strategies within existing residency structures.

The Kuehne et al. PHC Provider Advocacy Framework (12) frames advocacy as a collective responsibility of all PHC-oriented providers physicians, nurses, midwives, and community health workers specifically for PHC system strengthening. Using the PHCPI conceptual framework to structure advocacy targets (system and governance; inputs; service delivery), it proposes core competency domains: health policy and legislative advocacy; communication and media engagement; grassroots mobilization and community organizing; community partnership and collaboration; and teaching advocacy to others (12, 41). This framework uniquely positions advocacy within PHC system strengthening and explicitly addresses LMIC and UHC contexts. While not yet evaluated as a full longitudinal curriculum (Supplementary Appendix B), it provides the most globally applicable PHC-aligned competency architecture currently available.

### 3.4 Educational and training interventions

#### 3.4.1 Typology of interventions

The included sources describe a wide range of educational interventions. A four-type typology emerges from synthesis. The typology of educational and training interventions identified in the included literature is summarized in Table 3.

**Table 3:** Typology of advocacy and policy leadership training interventions identified in the included literature.

Type	Share of programs (approx.)	Duration	Core features	Example programs
Type 1—Didactic-only models	10%	1–6 h	Primarily lecture-based; limited interaction; minimal or no assessment; focus on basic advocacy or policy literacy.	Brief advocacy seminars; policy literacy lectures.
Type 2—Experiential project-based models	40%	Several weeks to 3–4 months	Student- or resident-led community advocacy projects; structured partnerships with community organisations; guided reflection on practice.	Child health advocacy curriculum (Majeed et al., 2020); community-based advocacy training using asset-based community development (Hufford et al., 2009); (42, 43)
Type 3—Longitudinal, integrated curricula	40%	6 months to 2 + years	Mandatory curricula embedded in residency/fellowship; combine didactic teaching, experiential projects, clinical rotations, mentorship and reflective practice.	Integrated Community Health and Child Advocacy Curriculum (Sieplinga et al., 2021); WONCA/Farley Health Policy Centre seven-month global curriculum for young family doctors (Amor et al., 2022); (31, 44)
Type 4—Leadership/governance-focused or transformational models	10%	6–12 months	Emphasis on professional identity formation; values-based leadership; practical systems skills.	South African family physician leadership and governance module (von Pressentin et al., 2024); (18)

#### 3.4.2 Pedagogical approaches

Across intervention types, several pedagogical elements emerge as consistently effective. The pedagogical approaches and effective elements identified across interventions are summarized in Table 4. Experiential and project-based learning is emphasized across contemporary programs: advocacy competencies are best developed through hands-on engagement with real or simulated advocacy challenges, including community projects, policy analysis assignments, health department rotations, and governance simulations. Longitudinal integration embedding advocacy across multiple years of training alongside clinical practice is associated with better outcomes and engagement than standalone sessions; Kuehne et al. (12), and Amor et al. (31), both specifically recommend longitudinal, hands-on, community-embedded learning. Mentorship and role modelling from physicians experienced in advocacy and from external mentors including policy makers, community leaders, and NGO directors is consistently considered an enabler, with structured mentorship programs showing higher learner engagement and confidence (32). Genuine community partnerships where communities are partners in designing and implementing advocacy projects rather than subjects of study are associated with stronger and more sustainable outcomes (43). More recent programs (the South African leadership module; WONCA global curriculum; newer paediatric programs) incorporate reflective practice, coaching, and transformative learning frameworks aimed at professional identity formation, resilience, and values clarification, rather than skill transmission alone. Programs that explicitly map content and assessments to defined competency frameworks consistently report clearer learning outcomes and more rigorous evaluation.

**Table 4:** Pedagogical approaches and effective elements identified in advocacy and policy leadership training interventions.

Approach	Effective elements (what works)
Experiential/project-based learning	Hands-on engagement with real or simulated advocacy challenges; community projects; policy analysis assignments; health department or policy rotations; governance simulations. Programs consistently report stronger competency development when learners “do” advocacy, not just hear about it.
Longitudinal integration	Embedding advocacy across multiple years, alongside clinical practice, rather than one-off workshops. Associated with better learner engagement and sustained outcomes. Examples Kuehne et al. (12); Amor et al. (31) recommend longitudinal, community-embedded, hands-on learning.
Mentorship and role modelling	Structured mentorship from physicians experienced in advocacy and from external mentors (policy makers, community leaders, NGO directors). Programs with intentional mentoring report higher learner engagement, confidence, and clearer understanding of “what advocacy looks like in real life” e.g., Chamberlain et al. (32).
Genuine community partnerships	Communities act as partners and co-designers of advocacy projects, not just “subjects of study.” Asset-based approaches and shared ownership are associated with stronger, more sustainable outcomes (e.g., Hufford et al. (43).
Reflective practice and transformative learning	Use of reflection, coaching, and values-based dialogue to support identity formation, resilience, and ethical clarity. Recent programs (e.g., South African leadership module, WONCA global curriculum, newer paediatrics curricula) emphasise “who I am as an advocate,” not only technical skills.
Competency-based design and assessment	Explicitly mapping content and assessments to defined competency frameworks (e.g., CanMEDS Health Advocate role, PHC-specific frameworks). These programs describe clearer learning outcomes, more rigorous evaluation, and better alignment between teaching, practice, and assessment.

#### 3.4.3 Core content areas

Understanding what is actually taught in advocacy and policy training programs is as important as knowing how it is structured. This section maps the substantive content domains identified across the 20 included sources, revealing areas of relative consensus and notable gaps, particularly for family medicine and PHC-oriented training Synthesis across the included sources identifies the following core content domains across advocacy and policy training programs: health policy and legislative advocacy (addressed in 100% of programs including policy literacy), encompassing understanding policy processes, legislative mechanisms, regulatory frameworks, and policy analysis; communication and media engagement (approximately 90% of programs), including op-ed writing, testimony preparation, social media, and persuasive communication; community engagement and organizing (approximately 85%), including asset-based community development, community partnership, social determinants frameworks, and grassroots organizing; research and evidence-based advocacy (approximately 70%), including using data and clinical observation as advocacy evidence; and equity and social justice (approximately 80%), including social determinants, health inequities, and anti-racist approaches. Notably fewer programs explicitly address health system structure, financing, governance, and district health planning core elements of the Kuehne PHC framework and the South African model representing a significant gap particularly for family medicine and PHC-oriented training globally. Figure 1 provides a visual summary of the six core content domains identified across the included sources. The distribution of advocacy and policy training interventions by specialty, region, and training level is summarized in Table 5.

**Figure 1:**
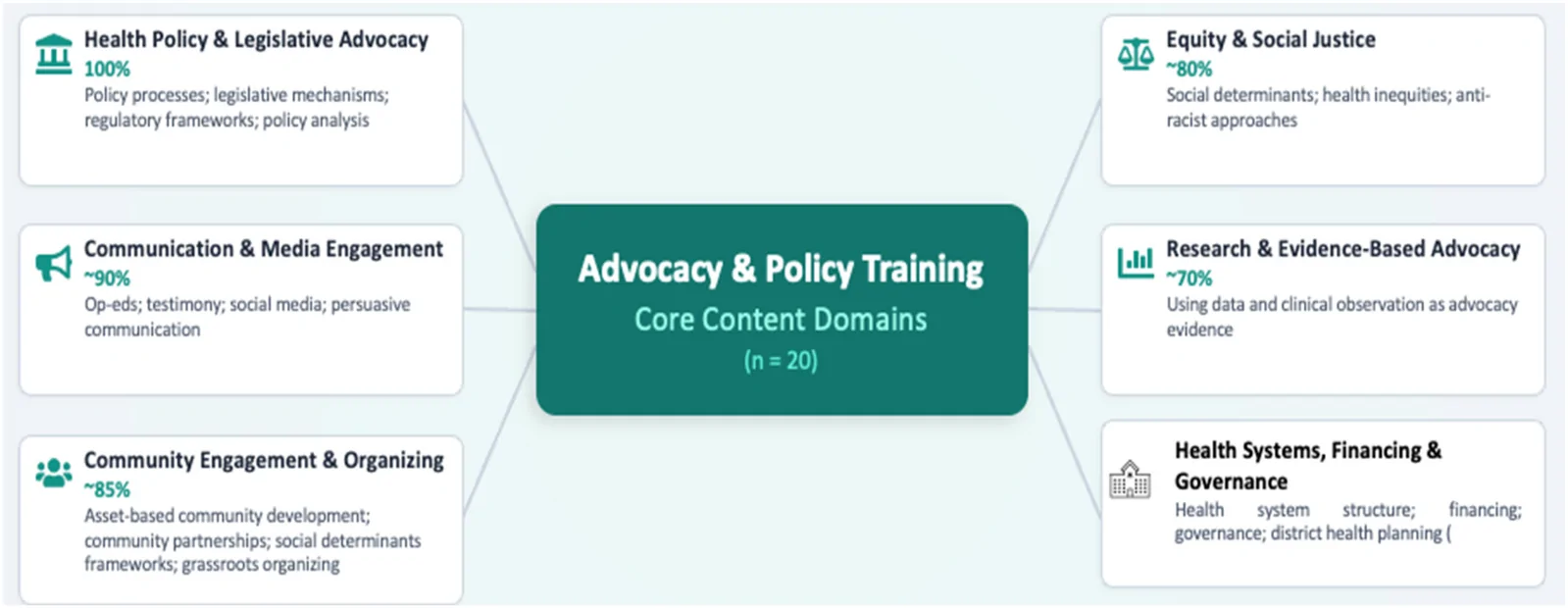
Core content domains of advocacy and policy training identified across 20 sources.

**Table 5:** Distribution of advocacy and policy training interventions by specialty, region, and training level.

Category	Primary distribution	*N* (approx.)	Key observations
Specialty	Paediatrics	∼35% of described curricula	Longstanding tradition of child health advocacy as professional responsibility
	Internal medicine	∼20%	Emphasis on policy literacy and population health
	Family medicine/PHC	∼25%	Emerging; South Africa and Turkey are exemplars but evaluation limited
	Interprofessional/other	∼20%	Includes nurses, midwives, CHWs; growing subset
Region	USA and Canada	>70% of published programs	Reflects publication practices, accreditation frameworks, and academic incentives
	South Africa, Turkey (LMIC/UMIC)	∼10%	Exemplary but under-documented in indexed literature
	Other LMIC regions	Very limited	Substantial under-representation; likely active programs in grey literature
Training Level	Postgraduate/GME residency	75%	Advocacy framed as applied professional competency once clinical identity established
	Undergraduate	10%	Missed opportunity for early, longitudinal competency development
	CPD/continuing education	10%	Growing but under-represented; Amor 2022 is a key example

### 3.5 Evidence of effectiveness and outcomes

#### 3.5.1 Kirkpatrick levels of evidence

Outcome measurement in the included sources follows the Kirkpatrick model (45). Level 1 (learner reaction) is rarely reported in detail. Level 2 (learning: knowledge, attitudes, self-efficacy) shows the strongest evidence: consistent, positive changes in advocacy knowledge, self-reported confidence, and expressed commitment to advocacy are documented across 75% of postgraduate programs in the core evidence base (14–16). Level 3 (behaviour: transfer to practice) shows moderate evidence at short-term follow-up (6–12 months), with some programs reporting increased advocacy activities including community involvement and policy engagement; however, longitudinal follow-up beyond three years is rare across all included sources. Level 4 (results: organizational, community, or policy-level changes) shows critically sparse evidence: only a handful of programs document organizational or policy changes attributable to training, and no included sources document sustained, system-level PHC changes linked to advocacy training a gap confirmed across all three prior systematic reviews (14–16). Evidence strength by Kirkpatrick level across the core sources is summarized in Table 6.

**Table 6:** Evidence strength by kirkpatrick level across core sources (*N* = 20).

Level	Outcome type	Evidence strength	Key sources	Critical gaps
Level 1	Learner reaction/satisfaction	W/Absent	Rarely reported	Insufficient data to draw conclusions
Level 2	Knowledge, attitudes, self-efficacy	Strong	Howell (14); Scott (15); Agrawal (16); Amor (31); Chamberlain (32)	Self-report bias; limited objective measurement
Level 3	Behavioural change in practice	Moderate (short-term only)	Sieplinga (44); Hufford (43); von Pressentin (18)	Near-absence of follow-up beyond 12 months; no LMIC data
Level 4	Organizational/system/policy impact	Very weak/Absent	No studies document PHC system-level change	Critical gap; prevents evidence-based advocacy for scaled investment

#### 3.5.2 Key effectiveness findings

Three main conclusions are supported by the evidence. First, structured advocacy training consistently improves learner-level knowledge and attitudes: positive changes in knowledge of advocacy concepts, self-reported confidence, expressed commitment, and attitude toward advocacy as a professional responsibility are documented across 75% of postgraduate programs. Second, experiential and longitudinal programs demonstrate stronger outcomes than didactic-only approaches: higher learner engagement, greater short-term behavioural change, and some evidence of institutional structure-building (advocacy committees, community partnerships) that outlasts the program. Third, community partnerships and mentorship are key enablers: programs that invest in genuine community partnerships and structured faculty or external mentorship consistently report higher learner competence, confidence, and sense of agency (31, 32, 43). Conversely, the evidence does not support conclusions about sustained behavioural change beyond 6–12 months, system- or policy-level impact, or cost-effectiveness and scalability none of which is addressed by any included source.

### 3.6 Equity, community engagement, and social accountability

The included literature demonstrates growing but variable attention to equity and social justice, organized across three tiers. Strong equity integration where equity is explicitly foregrounded with operational mechanisms is present in: the Kuehne PHC Framework, which frames advocacy as essential to equity and PHC system strengthening and emphasizes marginalized populations; the South African Leadership Framework, which embeds community-oriented primary care as a core family physician role within an equity-focused governance model; and newer paediatric programs incorporating anti-racist and anti-oppressive frameworks (44). Moderate equity integration where equity is a stated value, but mechanisms are not systematically embedded characterizes most generic CanMEDS-aligned programs. Limited or absent equity integration characterizes policy literacy curricula that focus on legislative process without connecting to structural health equity, and programs framing advocacy primarily as individual-level communication.

As synthesized in Supplementary Appendix C, community engagement models span a spectrum: from traditional approaches where communities are subjects of study with limited voice, to collaborative partnership models where communities co-design projects and community leaders participate in mentorship, to community-based participatory research and co-design approaches (advocated by Kuehne et al. (12), to social accountability mechanisms such as community health committees, citizen scorecards, and participatory health planning (central to PHC system strengthening but underrepresented in the GME literature). Explicit engagement with decolonial, anti-racist, and anti-oppressive frameworks remains rare in the advocacy and policy training literature, representing a significant gap especially in LMIC-oriented curricula where colonial legacies and structural inequities continue to shape health systems.

### 3.7 Barriers and enablers

As synthesized in Supplementary Appendix C, As synthesized in Supplementary Appendix C, barriers operate across multiple levels, spanning physicians’ engagement with policy advocacy as both users and providers of training, the delivery of that training, and institutional commitment to its integration, include: At the faculty level: limited advocacy training expertise and confidence; time constraints for mentorship-intensive programs; lack of clarity on competencies and assessment; insufficient institutional recognition or incentives. At the institutional level: curricular crowding; resource constraints for community partnerships and mentorship; variable accreditation mandates (programs with explicit mandates, such as CanMEDS in Canada, show higher implementation rates than those without); and institutional cultures prioritizing clinical excellence over advocacy. At the learner level: competing clinical demands and limited protected time; unclear advocacy career pathways; and limited role model exposure. Across HIC and LMIC contexts, as detailed in Supplementary Appendix C, specific barriers differ in form (e.g., bureaucratic complexity, siloed decision-making, leadership pathways and governance infrastructure) but converge on shared themes: governance access, political navigation, and interprofessional engagement.

In LMIC contexts, distinct barriers can exist in which advocacy itself carries structural risk, including political risk and safety concerns such as professional or personal consequences for challenging health system reforms, donor-driven health policy agendas that constrain domestic advocacy space, limited governance infrastructure as a mechanism for clinician input, and chronic resource constraints. These barriers (either separately, in some combination or collectively) undermine faculty development and mentorship programs, creating an environment where advocacy is actively discouraged. This has direct implications for training design, which must address both competency development and the political and institutional conditions under which advocacy can be safely and sustainably practised.

The South African and Turkish frameworks provide the strongest exemplars of how these barriers can be addressed through contextual adaptation and genuine stakeholder engagement (8, 18, 24). Supplementary Appendix C's synthesis of context-specific advocacy challenges and opportunities across HIC and LMIC settings provides the contextual grounding for the strategic priorities proposed below.

## 4 Discussion

### 4.1 Principal findings in context

This environmental scan synthesizes global evidence on advocacy and policy leadership frameworks, competencies, and educational interventions for family physicians and other primary care professionals, drawing on 20 core sources identified through a systematic scoping process and detailed in the accompanying appendices (Supplementary Appendix B: Competency Framework Comparison; Supplementary Appendix B: Framework and Program Descriptions; Supplementary Appendix C: Advocacy Challenges and Opportunities). The principal findings confirm and extend prior systematic reviews (14–16) while offering new contributions: explicit integration of PHC-oriented and LMIC-specific frameworks; a comparative synthesis of four major competency frameworks grounded in a standardized extraction protocol; and a strategic priorities framework positioned within the evidence base for international academic partnership in PHC transformation.

The convergence of all four major competency frameworks on six core domains health policy literacy, communication, community engagement, equity focus, leadership and governance, and interprofessional collaboration provides a robust empirical foundation for integrated curriculum design. Importantly, the evidence and the comparative analysis in Supplementary Appendix A together suggest that these domains must not be treated as separate modules but as interdependent dimensions of a unified professional competency, particularly for family physicians who must simultaneously navigate organizational authority, community accountability, and policy processes within PHC-oriented health systems (8, 12). The South African and Turkish frameworks exemplify how this integration can be operationalized within existing postgraduate training structures through context-responsive design, stakeholder engagement, and alignment with actual health system roles and governance structures.

### 4.2 PHC, UHC, the SDGs, and the case for systems-level investment

A central finding of this review is that the most relevant and transferable frameworks for LMIC and district health system contexts Kuehne et al. (12), Mash et al. (8), and von Pressentin et al. (18), explicitly anchor advocacy and policy leadership competencies to PHC system strengthening and UHC. The Astana Declaration (2) and the WHO Operational Framework for Primary Health Care (3) position PHC as the foundation for achieving UHC and SDG 3 (Good Health and Well-Being), and explicitly call for empowered health workers who can advocate for PHC-enabling policies, engage communities as co-creators of health, and exercise leadership within health systems. The Kuehne framework operationalizes this alignment by mapping advocacy responsibilities to PHCPI targets, governance and policy, health system inputs, and service delivery enabling advocacy training to be directly linked to PHC performance improvement goals (12, 41). The WHO Global Competency Framework for UHC and the WHO Global Competency and Outcomes Framework for Essential Public Health Functions further support a competency-based, interprofessional approach that links workforce development with equitable, population-oriented PHC functions (46, 47).

Equipping family physicians and primary care professionals with advocacy and governance competencies is not merely an educational aspiration but a systems-level investment in PHC infrastructure. When family physicians effectively advocate for equitable resource allocation, engage in district governance, build community accountability mechanisms, and advance health equity policies, the downstream effects extend to PHC service quality, workforce retention, community trust, and population health outcomes the very metrics by which UHC progress is measured. It is important to note that advocacy is best understood as one of several contributing inputs to meso- and macro-level policy change, operating alongside financing decisions, governance reforms, and broader political and social determinants; isolating its independent causal contribution to system-level outcomes is therefore inherently difficult, which explains, though does not excuse, the current absence of Level 4 evidence. The near-total absence of Kirkpatrick Level 4 evidence equally represents not only a research gap but a critical constraint on the field's ability to mobilize scaled investment and demonstrate return on PHC workforce development. Longitudinal, system-linked outcome studies are not optional enhancements but foundational requirements for the field to mature.

### 4.3 Community engagement, social accountability, and equity

A recurring tension across the included literature is between stated commitments to equity and community engagement and the actual operationalization of these values. As documented in Supplementary Appendix C, both HIC and LMIC contexts present distinct opportunities for advocacy leverage (e.g., professional and academic platforms in HICs, and community-embedded legitimacy and district health system integration in LMICs), alongside distinct constraints including governance access barriers, political risk, and interprofessional complexity. The synthesis highlights that advocacy is increasingly interprofessional and community-oriented across both contexts, requiring coalition-building, participatory approaches, and cultural humility as baseline competencies.

The social accountability model of community engagement central to PHC system strengthening offers a concrete pathway for deepening equity dimensions in advocacy training. Mechanisms such as community health committees, citizen scorecards, participatory health planning, and community-based monitoring of PHC performance link advocacy training directly to accountability structures within district health systems (12). Integrating these mechanisms from the outset of curriculum co-design as envisioned in the forthcoming multi-regional collaborative initiative would not only strengthen equity outcomes but provide learners with authentic, community-responsive advocacy experiences that build both competence and professional identity. For example, a district-based advocacy learning project could involve family medicine registrars, community representatives, and district managers jointly using local PHC performance data and community feedback to identify an access barrier, develop a feasible policy or service-improvement proposal, and agree on safe channels for presenting it within existing governance structures. Such work would position community members as co-designers, rather than recipients, of advocacy activity, while ensuring that the advocacy objective, evidence base, and level of political risk are locally determined. Explicit engagement with decolonial and anti-oppressive frameworks, while limited in the current evidence base, is essential for programs operating in LMICs and is an active priority for curriculum design going forward.

### 4.4 International academic partnership as a vehicle for PHC transformation

The evidence base reviewed here is predominantly produced in and for HIC settings, with publication patterns reflecting the structural advantages of North American academic medical education. As detailed in Supplementary Appendix C, governance access, political navigation, and interprofessional engagement are shared challenges across contexts but the specific entry points and constraints differ in form. The South African and Turkish exemplars demonstrate that LMIC-grounded, contextually adapted advocacy frameworks can be developed through rigorous consensus processes and operationalized within existing postgraduate training structures, producing competency frameworks arguably more directly relevant to the health system realities of family physicians in resource-constrained settings than their HIC counterparts (8, 18, 24).

North–South and South–South academic partnerships provide the structural mechanism to bridge this divide. Equitable partnerships characterized by shared governance, reciprocal learning, mutual benefit, and explicit attention to power dynamics can: facilitate contextual adaptation of competency frameworks; build faculty capacity in institutions where advocacy education expertise is limited; create shared platforms for curriculum development, evaluation, and dissemination; and generate the LMIC-relevant evidence base currently absent from the literature (5, 23). This approach aligns explicitly with SDG 17 and the Astana Declaration's call for whole-of-society approaches to PHC. The multi-regional collaborative co-design initiative informing this review which includes institutions from diverse HIC and LMIC contexts exemplifies the model, positioning this environmental scan as a shared foundation for evidence-informed, contextually adapted curriculum development.

### 4.5 Advocacy ethics and safety in politically constrained contexts

A critical and underexplored dimension is the ethics and safety of advocacy practice in politically constrained or restrictive environments. As noted in Supplementary Appendix C, political and institutional risk is present across both HIC and LMIC contexts, but manifests differently: in HICs, advocacy may challenge institutional priorities or professional hierarchies, while in LMICs, health advocacy can be politically sensitive where health reforms intersect with broader political or donor agendas, and clinicians may face institutional, professional, or political pushback. Most advocacy training programs documented in the literature assume stable governance environments where physician input is welcomed and the risks of advocacy are primarily professional rather than personal. Training should therefore include explicit preparation for safe and ethical advocacy. Core activities could include stakeholder and power mapping; assessment of potential professional, institutional, and personal risks; identification of legitimate internal, professional, community, and policy channels for action; protection of confidentiality; conflict-of-interest management; and reflection on when collective, indirect, or deferred advocacy is safer than public challenge. Scenario-based discussion for example, a clinician asked to publicly criticize a donor-supported service change that is restricting access for a marginalized community can help learners distinguish between advocacy goals, available evidence, potential harms, and contextually appropriate strategies.

The near-total absence of explicit ethics and safety frameworks for advocacy in constrained environments represents a significant gap. Developing such frameworks through consultation with ethicists, LMIC practitioners, legal scholars, and community representatives and integrating them explicitly into advocacy curricula is both an ethical imperative and a practical prerequisite for building genuinely contextually relevant and safe advocacy capacity. Failure to address these dimensions risks producing training that is theoretically comprehensive but practically inapplicable or actively harmful in constrained political environments.

### 4.6 Limitations

Several limitations should be noted. As an environmental scan conducted over an intensive two-week period, this review prioritizes breadth and conceptual mapping rather than exhaustive systematic coverage and does not claim to capture all published or unpublished work on advocacy and policy leadership training in primary care. The review is limited to English-language sources and to materials accessible through major academic and grey literature databases, likely underrepresenting innovations documented in non-English publications, internal reports, or locally disseminated materials, particularly from LMIC settings. This restriction may also have introduced language, publication, and indexing bias, favouring programs and frameworks developed in settings with greater capacity to publish in English-language journals or disseminate materials through internationally visible organizations. Consequently, locally developed curricula, internal program reports, and practice-based innovations from low- and middle-income countries may be underrepresented. The purposive selection of 20 core sources for detailed extraction further supports conceptual mapping rather than estimates of intervention prevalence or effectiveness. The evidence base is unevenly distributed across contexts, with a higher volume from HICs reflecting broader publication and indexing practices. Most included sources rely on self-report surveys for outcome measurement, and causal attribution of organizational or system changes to a training program remains methodologically challenging. The temporal scope (2004–2024) acknowledges but does not comprehensively review earlier conceptual contributions to physician advocacy, potentially limiting historical depth. Findings should be interpreted as analytically informed rather than prescriptive.

## 5 Strategic priorities and recommendations

The following eight strategic priorities emerge from synthesis of evidence, gaps, and patterns identified in this environmental scan. The strategic priorities, rationale, actions, and timelines are presented in Table 7. They are organized across short-term (0–2 years), medium-term (2–5 years), and long-term (5+ years) horizons, and are presented as a strategic menu rather than a sequential implementation plan. For collaborative curriculum co-design, Priorities 2, 6, and 8 are most immediately actionable; Priorities 1, 4, and 5 are medium-term institutional enablers; and Priorities 3 and 7 are longer-term investments essential for sustainability and system-level impact.

**Table 7:** Strategic priorities: rationale, actions, and timelines.

Priority	Horizon	Rationale	Key actions	Expected output
1. Develop a Unified Competency Framework	Medium-term (2–5 yrs)	Four frameworks identified; integration into a unified, globally applicable yet locally adaptable framework would facilitate coherence, benchmarking, and accreditation development (Supplementary Appendix B)	International working group (20–25 members; HIC + LMIC educators, practitioners, community representatives, accreditation bodies); consensus process; open access framework with implementation guidance	Internationally endorsed competency framework; operationalization guidance for diverse contexts; ongoing refinement platform
2. Develop and Pilot Context-Adapted Longitudinal Curricula	Short-term (0–2 yrs)	Near-absence of evaluated LMIC-specific postgraduate curricula; contextual adaptation, not transplantation, essential (Supplementary Appendix B)	Select 3–5 LMIC partner institutions across regions; participatory curriculum design; longitudinal integrated model (6–12 months); evaluation using Kirkpatrick Levels 1–3; document barriers and enablers	3–5 piloted, evaluated curricula available for scaling; implementation science insights on context-adapted training
3. Fund Longitudinal Studies Linking Training to System Outcomes	Long-term (5 + yrs)	Zero included studies document system-level PHC changes from training; critical barrier to scaled investment and policy reform	2–3 multi-year cohort studies (5–7 year timelines); outcomes at individual, organizational, and health system levels; mixed methods; comparison groups; diverse settings	Robust evidence linking training to sustained behaviour change and system-level outcomes; publications; evidence base for policy reform
4. Establish International Network for Advocacy Training Exchange	Medium-term (2–5 yrs)	Programs emerging but fragmented and poorly connected; networks would accelerate learning, resource sharing, and capacity building (SDG 17 alignment)	International consortium through WONCA, Starfield Summit, African Forum for Primary Care; digital platform for sharing curricula and materials; mentorship pairings; annual summit; published case studies	Active international network with 20–30 member institutions; shared resources; annual summit; growing evidence base
5. Integrate Competencies into Accreditation Standards	Medium-term (2–5 yrs)	Programs with explicit accreditation mandates show higher implementation rates; accreditation drives institutional accountability	Engage ACGME, RCPSC, national medical councils, nursing/midwifery bodies; advocate for explicit competency inclusion; develop assessment tools and rubrics; build faculty development requirements	Updated accreditation standards; assessment tools; evidence on implementation effectiveness
6. Build Faculty Development and Capacity	Short-term (0–2 yrs)	Many faculty lack training in advocacy teaching; systemic support essential to scale curricula	Develop workshops, online modules, and structured mentorship on: teaching advocacy, facilitating community partnerships, assessing skills, addressing political risk; train-the-trainer models; institutional incentives	Accessible faculty development resources; trained faculty cohort; train-the-trainer capacity
7. Conduct Implementation Science Research	Medium-term (2–5 yrs)	Limited context-specific research on enablers and barriers; implementation science would identify modifiable factors and effective strategies	2–3 mixed-methods implementation studies in diverse settings (HIC residencies, LMIC district programs, CPD); use CFIR framework; test implementation strategies; document processes and costs	Implementation science evidence; modifiable barrier identification; implementation toolkit for diverse settings
8. Develop Ethics and Safety Frameworks for Constrained Contexts	Short-term (0–2 yrs)	Near-total absence of advocacy ethics and safety frameworks, particularly for LMIC and politically constrained environments (Supplementary Appendix C)	Convene working group (ethicists, LMIC practitioners, legal scholars, community representatives); develop: ethical frameworks, risk assessment tools, collective safety strategies, guidance on advocacy in constrained environments; integrate into curricula from the outset	Published ethical frameworks and decision-making tools; integration into curricula and faculty development; guidance for global implementation
Establish a multi-country longitudinal evaluation platform	Long term (5 + years)	Current evidence rarely assesses outcomes beyond learner-level change and is especially limited in LMIC settings. Advocacy is one contributing input to not the sole cause of policy and health-system change.	Develop a prospective cohort across HIC, MIC, and LIC institutions, using common core and locally adapted measures of learner outcomes, advocacy practice, organizational capacity, community partnerships, and contribution to PHC strengthening. Align measures with WHO UHC and Essential Public Health Functions competency/outcomes frameworks	Generates equitable, LMIC-relevant longitudinal evidence on how training contributes to PHC functions, policy processes, and organizational capacity.

## 6 Conclusion

This environmental scan synthesizes global evidence on advocacy and policy leadership frameworks, competencies, and educational interventions for family physicians and primary care professionals, drawing on 20 core sources published between 2004 and 2024 and detailed across four supplementary appendices. The evidence confirms that advocacy and policy leadership training for primary care is evolving from *ad hoc*, values-based approaches toward competency-based frameworks aligned with PHC system strengthening but remains critically underdeveloped for LMIC and district health system contexts.

Four foundational competency frameworks CanMEDS, the South African District Health System Family Physician Leadership Framework, the Turkish Family Medicine Health Advocacy Competency Set, and the Kuehne PHC Provider Advocacy Framework converge on six core domains: health policy literacy, communication, community engagement, equity focus, leadership and governance, and interprofessional collaboration. Educational interventions that are longitudinal, experiential, community-engaged, and grounded in genuine partnership consistently demonstrate stronger outcomes at the learner level, though evidence for sustained behavioural change and organizational or system-level impact remains sparse across all contexts. Major gaps include the near-absence of fully evaluated, LMIC-specific postgraduate curricula; virtually no studies documenting policy-level system changes attributable to advocacy training; underexplored integration of equity, social accountability, and community engagement; and limited explicit attention to advocacy ethics and safety in politically constrained environments.

The eight strategic priorities proposed in this review spanning curriculum development, faculty capacity building, ethics and safety frameworks, international network building, accreditation reform, implementation science research, and longitudinal outcome studies provide a structured roadmap for advancing advocacy and policy leadership capacity in primary care globally. Critically, these priorities are embedded within a model of equitable, reciprocal international academic partnership that positions shared evidence synthesis as the foundation for collaborative curriculum co-design, knowledge exchange, and mutual capacity building across diverse health system contexts. This approach reflects the spirit of SDG 17, the Astana Declaration, and the growing global consensus that PHC transformation requires not only technical solutions but the coalitions, governance structures, and professional competencies to translate evidence into action at every level of health systems worldwide.
