# Supplementary material for: Advocacy and policy leadership training for primary care: a global environmental scan of competency frameworks, interventions, and strategic priorities
Source: Front Med (Lausanne). 2026 Sep 30;13:1894165. doi: 10.3389/fmed.2026.1894165 (PMC13662263; doi:10.3389/fmed.2026.1894165)
Supplement: Supplementary file 1 [file Datasheet1.pdf]

Appendix A
Competency Framework Comparison Table: Comparison of CanMEDS, South African, Turkish, and Kuehne frameworks with competency domains, definitions, and operationalization**.**

Framework	Primary Orientation	Core Competency Domains	How Advocacy Is Defined	How Policy Literacy Is Addressed	Leadership / Governance Focus	Degree of Operationalization
**CanMEDS Health Advocate (Canada)**	Generic, cross-specialty professional framework	• Individual patient advocacy<br>• Determinants of health<br>• Community engagement<br>• Health system and policy contribution	Advocacy framed as responding to patient and population health needs and supporting system improvement	Implicit; policy engagement acknowledged but not broken into analytic or procedural competencies	Implicit; leadership embedded across multiple CanMEDS roles but not explicitly tied to governance functions	**Low** – high-level role description; limited guidance for curriculum design, teaching strategies, or assessment
**South African FP District Health System Leadership Framework**	Family medicine–specific; district health system–anchored	• Clinical governance<br>• Community-oriented primary care (COPC)<br>• Leadership and teamwork<br>• Health system navigation<br>• Advocacy and influence	Advocacy embedded within relational and system-level roles; focused on influencing services, governance, and equity	Explicit; includes understanding of district health systems, policy environments, financing, and governance structures	Central; leadership framed as influence, stewardship, and system navigation rather than management alone	**High** – clearly articulated roles, domains, and skills; operationalized through longitudinal curricula and reflective, practice-based pedagogy
**Turkish Family Medicine Health Advocacy Competency Framework (Delphi-derived)**	Family medicine–specific; residency training focus	• Patient and community advocacy<br>• Health promotion<br>• Communication and ethics<br>• Professional responsibility<br>• System awareness	Advocacy defined as an integrated professional role spanning patient, community, and system levels	Explicit but bounded; focuses on understanding policy and system context relevant to family medicine practice	Moderate; leadership overlaps with professional and advocacy roles but governance is not the primary organizing principle	**Moderate–High** – competency statements, teaching methods, and assessment approaches identified through expert consensus
**Kuehne PHC Provider Advocacy Framework**	PHC system strengthening; global and LMIC-oriented	• Policy and legislative advocacy<br>• Community mobilization<br>• Communication and media<br>• Equity and social accountability<br>• Interprofessional collaboration	Advocacy framed as a responsibility of PHC providers to strengthen health systems and advance equity	Central; strong emphasis on policy cycles, financing, governance, and legislative processes	Strong; leadership embedded in collective, system-level action across PHC teams and communities	**Moderate** – conceptually robust with detailed advocacy pathways; pedagogical guidance provided but limited empirical evaluation

Summary

Across frameworks, advocacy is universally endorsed as a core professional responsibility, but the depth and function of that endorsement vary substantially. Generic frameworks such as CanMEDS provide essential normative grounding but remain abstract. In contrast, the South African, Turkish, and Kuehne frameworks progressively operationalize advocacy by integrating policy literacy and leadership into real health system roles, particularly within primary health care and district-level contexts. Notably, LMIC-anchored and PHC-oriented frameworks are more likely to treat advocacy, leadership, and policy literacy as interdependent competencies, reflecting the practical realities of health system strengthening rather than discrete educational silos.
