# Supplementary material for: Advocacy and policy leadership training for primary care: a global environmental scan of competency frameworks, interventions, and strategic priorities
Source: Front Med (Lausanne). 2026 Sep 30;13:1894165. doi: 10.3389/fmed.2026.1894165 (PMC13662263; doi:10.3389/fmed.2026.1894165)
Supplement: Supplementary file 2 [file Datasheet2.pdf]

Appendix B

Specific Frameworks and Program Descriptions: Detailed descriptions of South African district leadership model, Turkish FP competencies, Kuehne PHC framework, and implementation examples

Framework	Health System Orientation	Core Focus and Competency Emphasis	How Advocacy Is Conceptualized	Implementation Examples	Strengths for Training Design	Key Limitations / Gaps
**South African Family Physician District Leadership & Governance Framework** (Mash et al.; von Pressentin et al.)	LMIC; district health system–anchored PHC	• Clinical governance<br>• Community-Oriented Primary Care (COPC)<br>• Leadership and teamwork<br>• Health system navigation<br>• Equity and service improvement	Advocacy embedded as *internal advocacy* exercised through leadership, relationships, and governance roles within the district health system	• Longitudinal postgraduate modules in family medicine<br>• Registrars embedded in district health teams<br>• Participation in clinic governance, service planning, budgeting<br>• Group coaching, reflective practice, identity formation	• Highly contextualized to PHC realities<br>• Integrates advocacy, leadership, and policy literacy<br>• Aligns training with real system roles and authority	• Context-specific to South Africa<br>• Limited formal outcome evaluation to date<br>• Transferability requires adaptation to other systems
**Turkish Family Medicine Health Advocacy Competency Framework** (Demirören & Başer)	Upper-middle-income country; residency training context	• Patient and community advocacy<br>• Health promotion and prevention<br>• Communication and ethics<br>• Professional responsibility<br>• Health system awareness	Advocacy defined as a core professional competency spanning patient, community, and system levels	• Delphi-derived competency set for family medicine residency • Curriculum mapping within existing training<br>• Teaching via case discussions, community projects, reflection<br>• Assessment through observed performance and reflective tools	• Operationalizes advocacy into teachable competencies<br>• Feasible within existing residency structures<br>• Bridges conceptual roles and curriculum design	• Limited published data on implementation outcomes<br>• Leadership and governance less explicitly foregrounded
**Kuehne Primary Health Care Advocacy Framework**	Global; explicit LMIC and PHC system strengthening orientation	• Policy and legislative advocacy<br>• Health system financing and governance<br>• Community mobilization and social accountability<br>• Communication and media engagement<br>• Interprofessional collaboration	Advocacy framed as a collective responsibility of PHC providers to strengthen health systems and advance equity	• Short courses and capacity-building programs<br>• Advocacy toolkits and planning guides<br>• Community and policy engagement exercises<br>• Applied across diverse PHC reform contexts	• Explicitly links advocacy to PHC system strengthening<br>• Strong policy literacy and system focus<br>• Adaptable across professions and settings	• Largely conceptual<br>• Limited empirical evaluation of longitudinal curricula<br>• Less guidance on integration into formal residency programs

Summary

Together, these frameworks illustrate a progression from clinically embedded, governance-oriented advocacy (South Africa), to competency-based residency training (Turkey), to system- and policy-facing PHC advocacy (Kuehne). While differing in scope and maturity, all three move beyond generic definitions and provide concrete pathways for integrating advocacy, policy literacy, and leadership into primary care training. Importantly, they emphasize *adaptation rather than transplantation*, underscoring that effective advocacy education must align with local health system structures, professional roles, and political contexts.
