# Supplementary material for: Advocacy and policy leadership training for primary care: a global environmental scan of competency frameworks, interventions, and strategic priorities
Source: Front Med (Lausanne). 2026 Sep 30;13:1894165. doi: 10.3389/fmed.2026.1894165 (PMC13662263; doi:10.3389/fmed.2026.1894165)
Supplement: Supplementary file 3 [file Datasheet3.pdf]

Appendix C
Advocacy Challenges and Opportunities for Family Physicians and Primary Care Professionals Across Health System Contexts: A Synthesis of key advocacy challenges and opportunities faced by family physicians and primary care professionals across diverse health system contexts

Dimension	HIC Contexts	LMIC Contexts	Implications for Advocacy Training	Core Advocacy Competencies Required
**Systems and Governance Constraints**	Primary care often has limited formal representation in health system governance, with leadership pathways fragmented across institutions, regions, or professional bodies. Family physicians may face bureaucratic complexity and siloed decision-making structures that constrain system-level advocacy.	Formal leadership pathways for primary care professionals are often underdeveloped, particularly at district or subnational levels. Family physicians may practice in resource-limited settings with minimal governance infrastructure, limiting access to policy forums.	Training should build competencies in **health system navigation, governance literacy, and strategic engagement**, enabling advocacy within both complex institutional environments and resource-constrained systems.	• Health system literacy and governance understanding<br>• Ability to navigate multi-level policy and administrative structures<br>• Strategic positioning of primary care perspectives within system decision-making<br>• Policy analysis and agenda-setting skills
**Political and Institutional Risk**	Advocacy may challenge established institutional priorities, funding models, or professional hierarchies. Family physicians may encounter resistance when advocating for equity-oriented reforms, scope-of-practice changes, or PHC investment.	Health advocacy can be politically sensitive, particularly where health reforms intersect with broader political or donor agendas. Clinicians may face institutional, professional, or political pushback.	Training should include **risk assessment, ethical advocacy, and political acumen**, preparing physicians to engage constructively while managing professional and institutional constraints.	• Political acumen and contextual analysis<br>• Ethical advocacy and professional accountability<br>• Risk assessment and mitigation strategies<br>• Negotiation and constructive engagement with power structures
**Opportunities for Advocacy Leverage**	Family physicians often have access to professional organizations, academic platforms, and policy consultation mechanisms that can amplify advocacy efforts at regional or national levels.	Family physicians and PHC teams are frequently embedded in underserved communities and district health systems, providing legitimacy and urgency for advocacy related to access, financing, workforce, and service delivery.	Training should emphasize **leveraging positional authority, professional networks, and community credibility** to influence policy and system change across contexts.	• Strategic communication and persuasive advocacy<br>• Use of evidence and data to support policy arguments<br>• Leveraging professional and community networks<br>• Leadership and coalition-building
**Interprofessional and Community Dimensions**	PHC is increasingly delivered through interprofessional teams, requiring advocacy that aligns diverse professional perspectives and engages patients and communities in system improvement.	PHC delivery often involves diverse cadres, including nurses, midwives, community health workers, and informal providers, necessitating inclusive and coalition-based advocacy approaches.	Training should develop **interprofessional advocacy skills**, community engagement strategies, and coalition-building competencies that reflect team-based PHC delivery.	• Interprofessional collaboration and team-based advocacy<br>• Community engagement and participatory approaches<br>• Cultural humility and equity-oriented practice • Facilitation and consensus-building skills

Summary

This table synthesizes shared and context-specific advocacy challenges and opportunities for family physicians and primary care professionals across high-income and low- and middle-income settings. While governance constraints differ in form complex institutional structures in HICs and limited formal leadership pathways in LMICs, both contexts require strong system navigation and policy engagement skills. Political and institutional risks are present across settings, underscoring the need for ethical judgment, political acumen, and risk-aware advocacy. Opportunities for influence also vary, with professional and academic platforms more prominent in HICs and community-embedded legitimacy central in LMICs. Across contexts, advocacy is increasingly interprofessional and community-oriented, requiring collaboration, coalition-building, and participatory approaches. Overall, the synthesis highlights the importance of adaptable, competency-based advocacy training that is responsive to diverse health system realities rather than context-specific prescriptions.
